# Synthesized Zeolite Based on Egyptian Boiler Ash Residue and Kaolin for the Effective Removal of Heavy Metal Ions from Industrial Wastewater

**DOI:** 10.3390/nano13061091

**Published:** 2023-03-17

**Authors:** Ahmed H. Ibrahim, Xianjun Lyu, Amr B. ElDeeb

**Affiliations:** 1 College of Chemical and Biological Engineering, Shandong University of Science and Technology, Qingdao 266590, China; 2 Mining and Petroleum Department, Faculty of Engineering, Al-Azhar University, Cairo 11884, Egypt

**Keywords:** boiler ash residue, wastewater treatment, alkaline fusion, hydrothermal processing, zeolite, heavy metal ion removal, adsorption, mechanism

## Abstract

The increase of global environmental restrictions concerning solid and liquid industrial waste, in addition to the problem of climate change, which leads to a shortage of clean water resources, has raised interest in developing alternative and eco-friendly technologies for recycling and reducing the amount of these wastes. This study aims to utilize Sulfuric acid solid residue (SASR), which is produced as a useless waste in the multi-processing of Egyptian boiler ash. A modified mixture of SASR and kaolin was used as the basic component for synthesizing cost-effective zeolite using the alkaline fusion-hydrothermal method for the removal of heavy metal ions from industrial wastewater. The factors affecting the synthesis of zeolite, including the fusion temperature and SASR: kaolin mixing ratios, were investigated. The synthesized zeolite was characterized by X-ray diffraction (XRD), Fourier transform infrared spectroscopy (FTIR), scanning electron microscopy (SEM), particle size analysis (PSD) and N_2_ adsorption-desorption. The SASR: kaolin weight ratio of 1:1.5 yields faujasite and sodalite zeolite with 85.21% crystallinity, which then shows the best composition and characteristics of the synthesized zeolite. The factors affecting the adsorption of Zn^2+^, Pb^2+^, Cu^2+^, and Cd^2+^ ions from wastewater on synthesized zeolite surfaces, including the effect of pH, adsorbent dosage, contact time, initial concentration, and temperature, have been investigated. The obtained results indicate that a pseudo-second-order kinetic model and Langmuir isotherm model describe the adsorption process. The maximum adsorption capacities of Zn^2+^, Pb^2+^, Cu^2+^, and Cd^2+^ ions onto zeolite at 20 °C were 12.025, 15.96, 12.247, and 16.17 mg·g^−1^, respectively. The main mechanisms controlling the removal of these metal ions from aqueous solution by synthesized zeolite were proposed to be either surface adsorption, precipitation, or ion exchange. The quality of the wastewater sample obtained from the Egyptian General Petroleum Corporation (Eastern Desert, Egypt) was highly improved using the synthesized zeolite and the content of heavy metal ions was significantly reduced, which enhances the utilization of the treated water in agriculture.

## 1. Introduction

As a semi-arid region, Egypt suffers from water shortages, and the gap between water supply and water demand increases gradually with the rapid increase of the population. At the same time, Egypt’s Vision 2030 for sustainable socioeconomic development is based on the expansion of industrial and agricultural projects, which require a larger supply of water resources, and at the same time, the processing and utilization of industrial wastes to reduce their environmental effects [1,2]. The ability to increase current water resources from the River Nile became more difficult after the construction of the El-Nahda Dam in Ethiopia, and therefore, a feasible solution to fill this gap depends on the reuse and treatment of wastewater [3].

According to statistical assessments performed for 37 companies in Egypt, about 50% of those companies release their effluents of wastewater, only some of which are treated, of approximately 1.3 billion cubic meters (BCM)/year into the public sewage system in violation of Egyptian environmental law [4,5]. This leads to the deterioration of the sewage network, especially because industrial effluent contains massive loads of toxic materials, which increases the cost and environmental risks of sludge treatment and disposal [6,7]. Therefore, industrial wastewater is considered one of the world’s most serious global environmental problems today. The most prevalent hazardous metals present in industrial effluent are lead (Pb), cadmium (Cd), chrome (Cr), and nickel (Ni) [8]. These metals have been shown to be crucial for human health at low concentrations, but at elevated levels, they may be dangerous and seriously injure the liver, lungs, kidneys, and central nervous system. Therefore, the removal of heavy metal ions from industrial wastewater before releasing them into the environment is a challenge that requires immediate resolution [9].

Many techniques, including chemical precipitation [10,11], carbon adsorption [12,13], coagulation [14,15], membrane filtration [16,17], ion exchange, as well as adsorption [18,19], have been suggested for removing heavy metal ions from wastewater. Among these methods, the most direct and effective approach is adsorption because it is inexpensive, effective, highly selective, and insensitive to harmful chemicals [20]. Thus, developing an affordable, accessible, and highly effective adsorbent for removing hazardous metal ions presented in wastewater has become necessary. Utilization of solid industrial wastes by reusing them in the manufacturing of useful materials used for environmental purposes has been the subject of several recent studies and is one of the most crucial issues for maintaining sustainable development [21]. Kaolin has been widely used in many industrial applications due to its desirable physical and chemical properties, especially particle size, morphology, color, softness, non-abrasiveness, and chemical durability [22]. Moreover, its excellent physico-chemical surface properties (e.g., relatively low exchange and adsorption capacities, etc.), enhances its use in many applications [23]. The chemical stability over a relatively wide range of pH increases the application of kaolinite in paints, ceramics, rubber, and paper industries. In addition, the dispersibility of kaolin particles in water enhances its use as an ideal adjuvant in the pigment industry and in the production of poly-aluminum chloride (PAC), which is used in wastewater treatment [24].

The solid wastes containing silica (SiO_2_) and alumina (Al_2_O_3_), including industrial waste forms [25,26,27,28,29,30] (i.e., paper sludge, coal fly ash, fly ash from heavy fuel oil (HFO) combustion, and oil shale ash) such as alumino-silicate clays [31,32,33,34,35] and biomass [36,37,38] could be converted into ecological adsorbents (zeolite). To date, several physico-chemical and solvothermal techniques, including the hydrothermal approach [39,40,41], alkali-fusion method [42,43,44], sol-gel method [45,46,47], microwave method [48,49,50,51], and alkali-leaching method [52,53,54,55], have been adopted and developed to produce synthetic adsorbents. Zeolites are crystal formations built on stiff anionic alumino-silicate structures with distinct pores or channels that link at cavities or cages [56]. These materials are favorable for adsorption processes due to their high cation exchange capacity (CEC), large surface area, good thermal stability, porosity, surface active functional groups, and nontoxicity [57,58,59].

Many researchers have studied the synthesis of zeolites from solid wastes to be used as an adsorbent for removing heavy metal ions from wastewater. New zeolite materials have been processed from fly ash, successfully eliminating heavy metal ions from aqueous solutions [60]. It was noted that zeolites synthesized based on oil shale ash by an alkaline hydrothermal process were effectively used to extract Pb^2+^ and Cd^2+^ metal ions [61]. Synthesized Na-A zeolite from class F fly ash (FA) and modified oil shale ash (MOSA) by alkaline fusion followed by refluxing was used as an adsorbent for lead, zinc, and chrome [9]. Fly ash hydrothermally modified with NaOH solution was utilized to synthesize zeolites for Cd^2+^ adsorption, which showed effective removal of Cd^2+^ from the wastewater source [62]. Additionally, [63] a novel prepared adsorbent from coal fly ash (CFA) and solid alkali (NaOH) using a low-temperature roasting method was used for Cd^2+^ removal. A zeolitic material that was prepared from coal fly ash (CFA) through NaOH fusion treatment, followed by hydrothermal processing, was applied to removing heavy metal ions such as Ni^2+^, Cu^2+^, Cd^2+^, and Pb^2+^ from the wastewater source [64]. The adsorption capacity of the synthesized Na-A zeolite based on the fusion and hydrothermal treatment of oil shale was evaluated by measuring the maximum removal efficiency of Cu^2+^, Ni^2+^, Pb^2+^, and Cd^2+^ from aqueous solutions [8]. A new composite material was formulated based on fly ash (FA), meta-kaolin (MK), and TiO_2_ to form a micro-porous zeolitic material with enhanced photocatalytic properties for the adsorption of methylene blue (MB) dye [65]. Amorphous alumino-silicate geopolymer adsorbent synthesized from fly ash and solid NaOH with a maximum Cd^2+^ adsorption capacity of 26.25 mg·g^−1^ has also been investigated [66]. The alkaline fusion-hydrothermal process was used to synthesize zeolites from Brazil oil shale ash. It was noted that the synthesized zeolites are composed of likewise mixed phases (Na-A zeolite, Na-X zeolite, hydroxy sodalite, and quartz) [67]. The synthesis of zeolite for wastewater treatment based on a mixture of sulfuric acid solid residue (SASR) obtained from the Egyptian Boiler ash as by-products and kaolin concentrate using the alkaline fusion-hydrothermal process has not previously been investigated, and needs further investigation.

This study aims to investigate the formulation of synthesized zeolite based on the previously obtained sulfuric acid solid residue [68] and kaolin concentrate using an alkaline fusion-hydrothermal process for using in wastewater treatment. The factors affecting the synthesis of the targeted type of zeolite and the absorption of metal ions from the industrial wastewater were thoroughly investigated.

## 2. Materials and Methods

### 2.1. Materials

The recycled sulfuric acid solid residue (SASR) is one of the main materials used in this study from which the targeted zeolite type has been synthesized. The used SASR was obtained as a by-product from previous work carried out by the research team on the Egyptian boiler ash. Firstly, vanadium was extracted from Egyptian boiler ash using salt-roasting, water-leaching techniques [69], that produced water-leaching solid residue (WLSR) as by-product. WLSR was used to extract high-purity nickel and zinc metal ions using a selective and cost-effective sulfuric acid leaching process [68]. The sulfuric acid solid residue (SASR) obtained as a by-product from processing WLSR is used in this investigation as a source for alumina and silica to produce the targeted zeolite type. Both physical and acidic pretreatment processes were carried out to enhance the physico-chemical properties of the used SASR. In the physical treatment process, the induced roll dry magnetic separator (Carpco, Model, MIH (13) 111-5, Outokumpu Technology, Inc., Jacksonville, Florida, U.S.A.) was used to capture impurities having magnetic properties (Iron) to reduce the consumption of the necessary chemicals to synthesize the targeted zeolite. The operation conditions of magnetic separation were adjusted at a magnetic field of 1.4 T (current to the electromagnetic coil 2 Ampere), a roll speed of 60 rpm, and a sample feeding rate of 0.9 kg/h. [70,71]. After that, 10 g of obtained magnetic-pretreated SASR was subjected to acidic leaching using 50 mL of 1 M HCl solution at 80 °C for 4 h in a Pyrex three-neck round-bottom flask equipped with a reflux condenser in a hemispherical heating mantle [67]. The acid treatment eliminated the remaining iron, which reduced the iron content to the allowed limit, making it suitable for the synthesis of the zeolite framework at a higher conversion efficiency.

The overall chemical compositions of the SASR before and after pretreatment was determined by the X-ray fluorescence (XRF) method. The as-received raw and acidic leached SASRs have a SiO_2_/Al_2_O_3_ weight ratio of 4.57 and 5.05, respectively. Compared with the chemical composition of fly ash used in previous works [3,4,5,6], the low SiO_2_/Al_2_O_3_ ratio of ash was more favorable to synthesize low silicon-to-aluminum zeolite. Therefore, it is necessary to add a source of Al_2_O_3_ to the reaction mixture to achieve the desired SiO_2_/Al_2_O_3_ molar ratio. The source of Al_2_O_3_ used in this study was obtained from Wadi Qena kaolinitic sandstone [72]. Attrition-scrubbing experiments were carried out using a “Denver 12” flotation cell to separate the kaolinite and quartz minerals, making the composition of the obtained kaolinite mineral suitable for the synthesis of the targeted zeolite. The attrition-scrubbing process was optimized with the following specifications: pulp density 50% solid (*w*/*w*), impeller speed 1800 rpm, and attrition time 15 min (at three steps, each was conducted after desliming on 45 µm sieving).

In this work, analytical-grade chemical reagents including ZnSO_4_·7H_2_O, Pb(NO_3_)_2_, CuSO_4_·5H_2_O, and CdCl_2_·H_2_O were used to prepare standard stock solutions of Zn^2+^, Pb^2+^, Cu^2+^, and Cd^2+^ by dissolving these salts in double-distilled water. The wastewater sample used in this study was obtained from location owned by the Egyptian General petroleum Corporation (EGPC), before being discharged. The wastewater sample was prepared by filtration on a cotton membrane to eliminate the suspended matter and oils.

### 2.2. Methods

#### 2.2.1. Synthesis of Zeolite

**Scheme 1:**
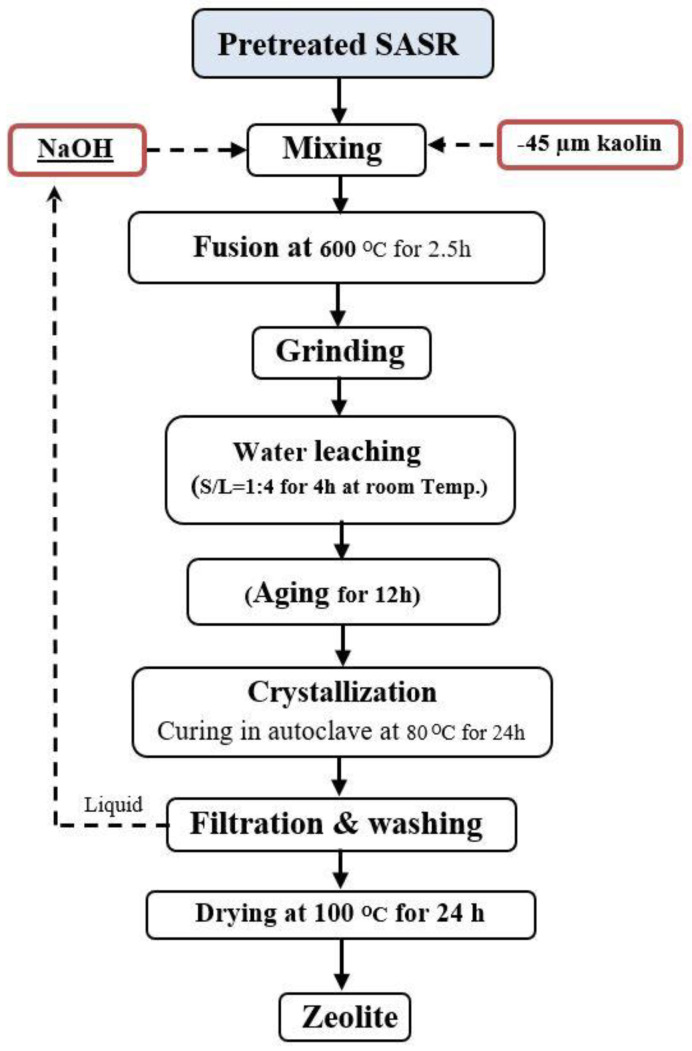
Schematic flow diagram for synthesized zeolite based on mixture of SASR and kaolin concentrate using the alkali-fusion method.

To produce the zeolite samples, the pretreated SASR was mixed with kaolin concentrate obtained from attrition scrubbing of Wadi Qena kaolinitic sandstone at weight ratios of 1:0, 1:2, 1:1.5, 1:1, 2:1, and 0:1, then sodium hydroxide was added to the mixture at weight ratios of 1:1.3. The mixture was then thoroughly ground using agate mortar for 20 min, and after this, the finally prepared mixture was fused in a muffle furnace (Nubertherm, Mod. N300/H, Lilienthal, Germany) at 600 °C for 2.5 h in a closed atmosphere at a rate of 10 °C·min^−1^ [26]. After the alkali fusion treatment procedure, the fused material was cooled and ground briefly with agate mortar and pestle for a few minutes, then agitated in distilled water using magnetic stirring at 1:4 g·mL^−1^ solid-liquid ratio, for 4 h at 500 rpm, then aged for 12 h at room temperature to obtain the precursor gel [8]. The obtained slurry was then sealed in a Teflon-lined autoclave and heated at 80 °C in a drying oven for 24 h [25]. Finally, the solid product was filtered out of the sealed slurry, washed with distilled water until the pH value of the filtrate was neutral, and then dried at 100 °C for 24 h. The physical composition of the synthesized product was characterized by XRD, FT-IR, and SEM-EDX. The schematic flow diagram of the synthesis process is shown in Scheme 1.

#### 2.2.2. Adsorption Experiments

Batch adsorption experiments were performed on the synthesized zeolite to examine its effectiveness in adsorbing Zn^2+^, Pb^2+^, Cu^2+^, and Cd^2+^ metal ions from the targeted wastewater sample. The effects of pH level, contact time, adsorbent dose, and initial metal ions concentration on the efficiency of the adsorption process were studied using 50 mL conical flasks. All experiments were carried out by mixing 25 mL of the prepared metal ions solutions containing various concentrations of metal ions (10−80 mg.L^−1^) with a given sorbent dose (0.1 g) at room temperature. The solution’s pH was adjusted using 0.1 M HCl and 0.1 M NaOH solutions. The suspension was then agitated on a rotary shaker to obtain homogenous and equilibrium concentrations of the metal ions at a speed of 200 rpm for 30 min. and pH range (3–11). Once the experiment time had finished, the adsorbent was removed from the metal ion solutions by centrifugation at 4000 rpm for 10 min. The concentrations of heavy metal ions in the solution were analyzed using Inductively Coupled Plasma Atomic Mass Spectrometry (ICP-MS, a Perkin Elmer ELAN model 9000, Waltham, MA, USA). The removal efficiency of a specific metal ion *R.E* (%), and the quantity of metal ions adsorbed at equilibrium per unit mass of the examined adsorbent ($q_{e}$, mg·g^−1^) were determined using Equations (1) and (2).

(1) $$R.E\left(\%\right)=\left[\frac{\left(C_{0}-C_{t}\right)}{C_{0}}\right]\times 100$$

(2) $$q_{e}=\frac{\left(C_{0}-C_{t}\right)\times V}{m_{s}}$$

where *R.E(%)* is the removal efficiency of a specific metal ion, $q_{e}$ is the uptake capacity (mg·g^−1^), $C_{0}$ is the initial metal ion concentration in solution (mg·L^−1^), $C_{t}$ is the equilibrium concentration at a specific time (mg·L^−1^), V is the solution volume (L), and m_s_ is the mass of the adsorbent (g).

#### 2.2.3. Materials Characterizations

The SASR samples before and after the treatment processes were characterized chemically by X-ray fluorescence (XRF) using a Shimadzu XRF analyzer (XRF-1800, 90 mA, 40 kV, Re anode, Kyoto, Japan). The changes in the mineralogical compositions of the SASR and the synthesized zeolite were examined using XRD (Analytical X-Ray Diffraction equipment model X” Pert PRO with Mono-chromator, Cu-Kα radiation (λ = 1.542 A) at 50 KV, 40 mA and scanning speed 0.02/s), and phase identification by PAN analytical X’Pert High Score Plus software. According to the diffraction peak intensity of the XRD patterns, Equation (3) was used to determine the crystallinity of synthesized zeolite.

(3) $$\mathrm{Crystallinity}\;(\%)=\left(\frac{\Sigma \;hights\;of\;XRD\;peaks\;of\;\mathrm{product}\;}{\Sigma \;hights\;of\;XRD\;peaks\;of\;\mathrm{stander}-\;\mathrm{zeolite}\;A}\right)\times 100$$

The Fourier Transform Spectrum of the synthesized zeolite samples was measured using a Perkin-Elmer infrared spectrometer, USA. The samples were produced using the KBr pellet technique, and then were scanned in transmission mode with a resolution of 4 cm^−1^ at a number wave range of 400 to 4000 cm^−1^. The Braunauere-Emmette-Teller (BET) method of multilayered adsorption was used to determine the specific surface area of the synthesized zeolite. Barret Joyner and Halenda (BJH) methods were used to determine the pore volume and average pore diameter. The sample was degassed for 12 h at 300 °C prior to the test, and the N_2_ adsorption-desorption measurement was done at the liquid nitrogen temperature of 77 °k. The average particle size of synthesized zeolite was measured using a particle size analyzer (Malvern Instruments Hydro 2000S Master Size, Malvern, UK). An X-ray Photoelectron Spectroscopy (XPS) spectrometer (Axis Ultra DLD-600W, Kratos, UK) was used to evaluate the composition of the loaded elements on the adsorbent. Inductively coupled plasma atomic mass spectrometry (ICP-MS, a Perkin Elmer ELAN model 9000, Waltham, MA, USA) was also used to accurately determine the concentration of metal ions in the used industrial wastewater samples before and after the adsorption process. Microstructural and elemental analyses of the synthesized zeolite were conducted using SEM (Tescan TS 5130MM) with an energy-dispersive X-ray (EDX) detector (prepared by Oxford Instruments, Abingdon, UK, active crystal area-50 mm^2^) in addition to a microanalysis system and YAG crystal as a backscattered electron (BSE) detector.

## 3. Results and Discussion

### 3.1. Characterization of Raw Materials and Synthesized Zeolites

The chemical analysis of the SASR after magnetic and acidic treatment processes is shown in Table 1, which indicates that these processes were effective and resulted in a high reduction in the Fe_2_O_3_ content (87.4%) after treatment. Quartz (SiO_2_), the major crystalline phase in the pretreated SASR, was not attacked by the HCl solution in the acidic treatment process. After fusion, the calcined mixture has a sufficient concentration of Al_2_O_3_ (27.81%) to produce zeolite.

**Figure 1:**
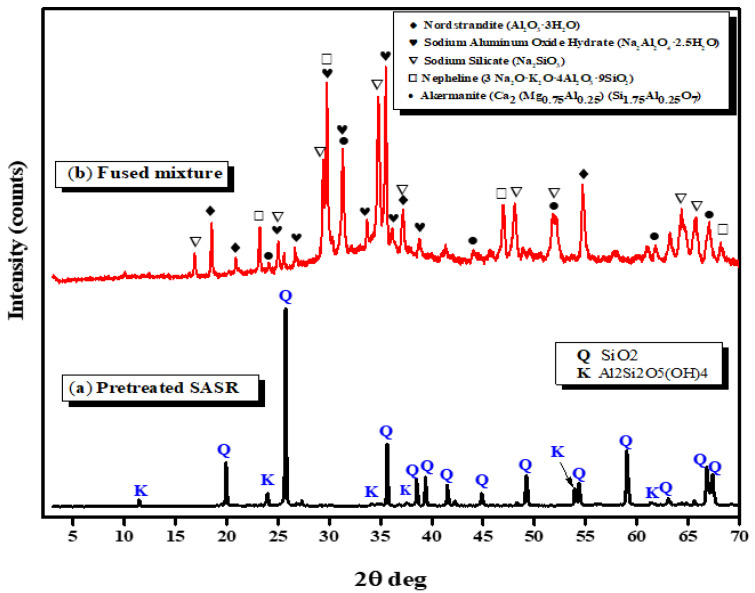
XRD patterns of the pretreated SASR (**a**), and the obtained fused mixture (1:1.5 SASR-Kaolin weight ratio) (**b**).

The X-ray diffraction patterns of the pretreated SASR and the fused mixture products are presented in Figure 1. The XRD pattern of pretreated SASR indicates that it is composed mainly of crystalline quartz and a minimum amount of alumino-silicate hydrate phase. It was noted that the aluminosilicate hydrate and quartz peaks in the fused mixture with a 1:1.5 SASR-kaolin weight ratio decreased, and new peaks, made up of sodium silicate (Na_2_SiO_3_) and sodium aluminium oxide hydrate (Na_2_Al_2_O_4_·2.5H_2_O), can be detected. This can be attributed to the fact that an appropriate NaOH concentration in the reaction mixtures works as an activator during fusion to create water-soluble silicate and aluminate salts and accelerates crystal growth during the crystallization process [25], according to Equations (4)–(6).

(4) $$\mathrm{NaOH}+x{\mathrm{Al}}_{2}O_{3}\;\times \;y{\mathrm{SiO}}_{2}\;\overset{\;\mathrm{Fusion}\;\mathrm{at}\;600\;{}^{\circ}C}{\to }{\mathrm{Na}}_{2}{\mathrm{SiO}}_{3}+{\mathrm{Na}}_{2}{\mathrm{AlO}}_{2}$$

(5) $$\mathrm{NaOH}\;(\mathrm{aq})+{\mathrm{Na}}_{2}\mathrm{Al}(\mathrm{OH}{)}_{4}\;(\mathrm{aq})+{\mathrm{Na}}_{2}{\mathrm{SiO}}_{3}\;(\mathrm{aq})\;\overset{Aging\;at\;25\;{}^{\circ}C}{\to }[\mathrm{Na}x({\mathrm{AlO}}_{2})y({\mathrm{SiO}}_{2})z\times \mathrm{NaOH}\times H_{2}O]\;(gel)$$

(6) $$\left[\mathrm{Na}x({\mathrm{AlO}}_{2})y({\mathrm{SiO}}_{2})z\;\times \;\mathrm{NaOH}\;\times \;H_{2}O]\;(gel)\;\overset{Crystalization\;80\;{}^{\circ}C\;}{\to }\;\mathrm{Nap}[({\mathrm{AlO}}_{2})p({\mathrm{SiO}}_{2})q]\times hH_{2}O\;(Crystal\;in\;Suspension\right)$$

**Figure 2:**
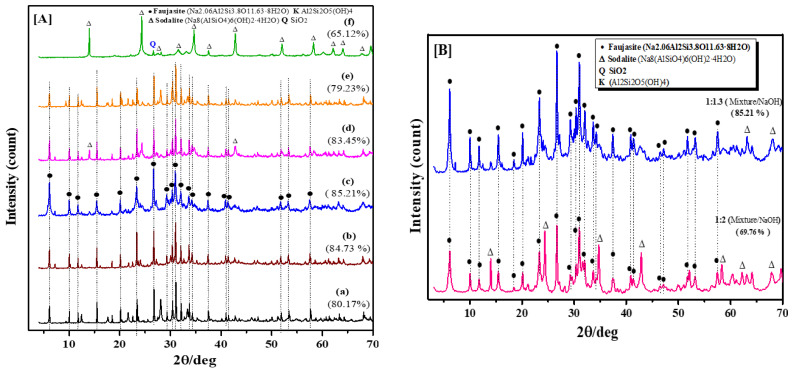
XRD Patterns of synthesized zeolite produced from mixture of SASR-Kaolin at weight ratios of (a) 1:0, (b) 1:2, (c) 1:1.5, (d) 1:1, (e) 2:1, and (f) 0:1 (**A**), XRD Patterns of synthesized products with different mass ratio of mixture-NaOH of 1:1.3 and 1:2 (**B**), where ●: Faujasite, Q: quartz, **∆**: Sodalite zeolite, and K: Al_2_Si_2_O_5_(OH)_4_.

Figure 2 illustrates the mineralogical structure of the produced synthesized zeolite by mixing pretreated SASR and kaolin mixtures at different weight ratios in the presence of NaOH (Mixture-NaOH mass ratio of 1:1.3). Figure 2A shows that Faujasite zeolite (Na_2_ Al_2_Si_3.8_O_11.63_·8H_2_O) was formed as the major mineral phase from SASR-Kaolin weight ratios of 1:0, 1:2, 1:1.5, 1:1, and 2:1. However, the intensity of its peaks increased with increases of the mass of kaolin, and a high rate of crystallinity (85.21%) can be obtained when the ratio of SASR to kaolin is 1:1.5, due to the stability of the zeolite composition. A percentage of sodalite zeolite (Na_8_(AlSiO_4_)_6_(OH)_2_·4H_2_O) groups also existed at a SASR-Kaolin weight ratio of 0:1. Figure 2B shows that the crystallinity of the synthesized zeolite decreased from 85.21% to 69.76% at a 1:2 mixture-NaOH weight ratio. However, when the alkalinity of the system is too high, a decline in crystallinity is caused. This can be attributed to one of the following two reasons: either the created zeolite spontaneously transforms into hydroxyl sodalite, which has a greater thermodynamic stability [26]; or the zeolite will dissolve in the hot alkali solution because of its metastable state [21,25].

**Figure 3:**
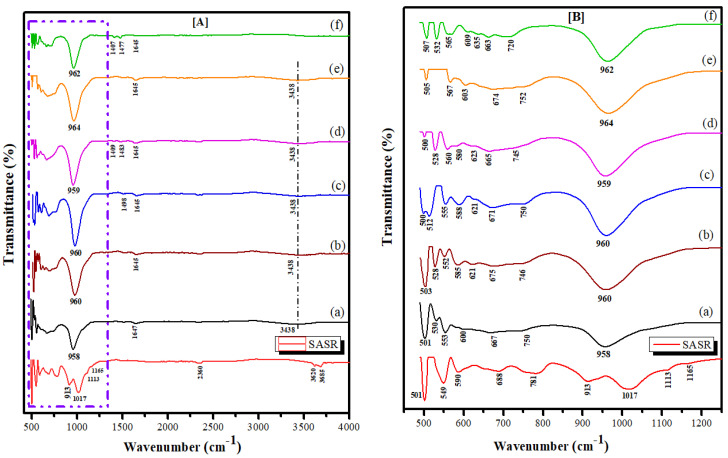
FTIR spectra of synthesized zeolite produced from SASR-Kaolin mixture at weight ratios of (a) 1:0, (b) 1:2, (c) 1:1.5, (d) 1:1, (e) 2:1, and (f) 0:1 (**A**), and detailed FTIR spectra in 1250–500 cm^−1^ wave number region (**B**).

The FTIR spectra of pretreated SASR and synthesized zeolite produced from SASR-Kaolin mixture at weight ratios of 1:0, 1:2, 1:1.5, 1:1, 2:1, and 0:1 are presented in Figure 3. Figure 3A shows that the characteristic peaks of the SASR were lost, and new peaks were identified, confirming the generation of new products. All samples display an band around 1645 cm^−1^ with a lower intensity peak, which can be attributed to the deformational vibrations of adsorbed water (O-H) molecules. The broad band of approximately 3438 cm^−1^ was caused by the surface structural hydroxyl groups and adsorbed water content, proving the hydrophilicity of the materials [26]. Figure 3B illustrates a clearer display of the two strongest zeolite bands, found at 420–500 cm^−1^ and 860–1230 cm^−1^, respectively [9,25]. The bands in the region 420–500 cm^−1^ were attributed to the bending vibration modes of T-O bonds (internal deformations of the zeolite) in the TO_4_ tetrahedral (T = Si/Al). The intensity of the initial band at 500 cm^−1^ increased until its lowest level when the mass ratio of SASR-Kaolin was 2:1. The bands in the region 500–650 cm^−1^ were associated with the vibrations of the double rings (4-membered ring and 6-membered ring). A new band was formed around 555 cm^−1^, which is the major secondary building unit of zeolite, that appeared with high intensity when the mass ratio of SASR-Kaolin was 1:1.5. The bands in pretreated SASR that defined the Al^IV^-O tetrahedron stretching vibrations at 781 cm^−1^ and 913 cm^−1^ were disappeared. A new observed band in the region 650–750 cm^−1^ was assigned to the symmetric stretching vibration of internal tetrahedral units (Si; Al)–O–(Si; Al). In addition, the tetrahedral asymmetric stretch vibrations peak of O–(Si; Al)–O were shifted from 1017 to 960 cm^−1^, which is considered the major band assigned to synthesized zeolite, with a high band intensity at a 1:1.5 mass ratio of SASR-Kaolin after hydrothermal modification. This observation can be explained by the fact that the Si-O and Al-O bonds of pretreated SASR were gradually broken during the hydrothermal process, which led to the cracking of a portion of the network structure made up of [SiO_4_] and [AlO_6_]. As a result, hydrothermal modification reduced polymerization degrees of [SiO_4_] and [AlO_6_] in the pretreated SASR [62].

**Figure 4:**
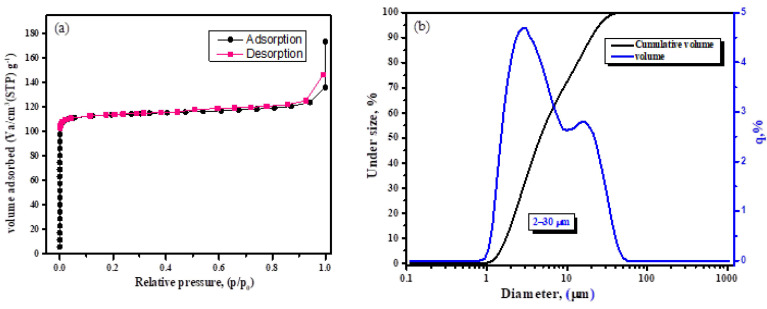
N_2_ adsorption-desorption isotherms (**a**), particle size distribution of the synthesized zeolite (**b**).

The N_2_ adsorption-desorption method was used to examine the surface characteristics of the synthetized zeolite, and the obtained results are presented in Figure 4a. The synthesized zeolite has a type IV isotherm and a type H3 hysteresis loop. The high adsorption at low relative pressures (P/P_0_ < 0.1) indicates that the structure of synthesized product are typical mesoporous materials [26]. Figure 4b presents the particle size distribution of the synthesized zeolite. According to the PSD, the synthesized zeolite had D_50_ and D_90_ (i.e., the median particle size that accounts for 50% and 90% of all particles, respectively) of around 5 and 20 µm, respectively. In the adsorption processes, the particle size distribution is of crucial importance because increasing the surface area of particles leads to increasing the number of active binding sites of the adsorbent, thus, improving the elimination of heavy metal ions from the targeted solution [73].

The surface characteristics of the synthesized zeolite are shown in Table 2. The BET analysis indicates that the specific surface area of zeolite increased from 75.46 to 473.54 cm^3^·g^−1^ at the 1:1.5 SASR-kaolin mass ratio, and then decreased to reach 62.84 cm^3^·g^−1^ at the 1:0 SASR-kaolin mass ratio. In addition, the pore volume and average pore size of the synthesized zeolite were 0.207 cm^3^·g^−1^ and 8.23 nm, respectively. The BET analysis revealed that the surface area of the synthesized zeolite was significantly larger than that obtained before in previous studies [26,62,65], enhancing its use as effective adsorbent. According to the previous observation, it can be confirmed that the synthesized zeolite based on the SASR-kaolin mixture at a mass ratio of 1:1.5 is recommended to be used in the following section of this work.

### 3.2. Optimization of Adsorption Behavior of Heavy Metal Ions on Synthesized Zeolite

#### 3.2.1. Effect of pH

**Figure 5:**
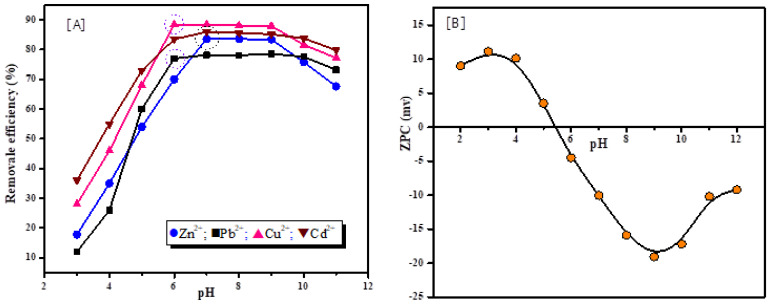
Effects of pH values on the adsorption efficiency of Zn^2+^, Pb^2+^, Cu^2+^, Cd^2+^ metal ions on the adsorbent surface (**A**), Point of zero charge (PZC) of synthesized zeolite (**B**).

One of the significant factors that influences the degree of ionization and speciation of the adsorbate, as well as the surface charges of the adsorbent during the adsorption process, is the pH value of the aqueous solution. Figure 5A presents the influence of pH values in the range from 3.0 to 11.0 on the adsorption of Zn^2+^, Pb^2+^, Cu^2+^, and Cd^2+^ metal ions using the synthetic zeolite. It was noted that when pH rises, the adsorption efficiency of metal ions increases. The removal efficiency of Zn^2+^ and Cd^2+^ raises from 17.8% to 83.8% and 36.01% to 85.98%, respectively, when the pH of the solution increases from 3 to 7. Similarly, the removal efficiency of Pb^2+^ and Cu^2+^ increases from 12.03% to 77.13% and from 28.11% to 88.48%, respectively, when the solution’s pH increases from 3 to 6. According to the obtained results, the recommended pH value for the highest removal of Zn^2+^ and Cd^2+^ is ≤7, while for the removal of Pb^2+^ and Cu^2+^, it is ≤6. The following equations explain the exchange process between metal ions and hydrogen ions.

X–OH + H_3_O^+^ → X–OH_2_^+^ + H_2_O (X = Si, Al, etc.) (7)

X–OH_2_^+^ + M^2+^ → X–O–M (M = Zn, Pb, Cu, Cd, etc.) (8)

X–OH + OH^−^ → X–O^−^+ H_2_O (9)

2(X–O^−^)+ M^2+^ → X–O–M–O–X (10)

Figure 5B presents that in the medium of high acidity, the surface of zeolite hydrated and acquired appositive charge due to excessive hydrogen ion (H^+^) (Equation (7)) and is partially replaced by metal ion (M^2+^) (Equation (8)). Hence, the uptake capacity of metal ions was reduced as a result of partial destruction of the zeolite structure and the competition (repulsive force) between cationic ions and H^+^ for the active adsorption sites (Si–OH$\begin{array}{c} +\\ 2 \end{array}$ and Al–OH$\begin{array}{c} +\\ 2 \end{array})$ on the adsorbent surface [8]. Contrarily, it was noted that when pH increases, the adsorption capacity of the adsorbent also increases, which can be attributed to the increase in the negative charge (Si–O^−^ and Al–O^−^) derived from the dissociation of hydroxyl group (Equation (9)) on the surface of adsorbent. As a result, the electrostatic attraction between metal ions and solid surface sites also increases (Equation (10)) [62]. After the adsorption process and the rise of the pH value, cationic ions can directly react with OH^−^ to generate hydrated heavy metal cations M(OH)**^+^** according to Equation (11), which leads to an increase in the concentration of H^+^ ions. Then, the hydrated M(OH)^+^ react with the hydroxyl group to form precipitation compounds on the surface of the adsorbate (Equation (12)) [19].

M^2+^ + H_2_O → M(OH)^+^ + H^+^ (11)

X-OH + M(OH)^+^ ⇄ XM(OH)_2_ (12)

#### 3.2.2. Effect of Adsorbent Dosage

**Figure 6:**
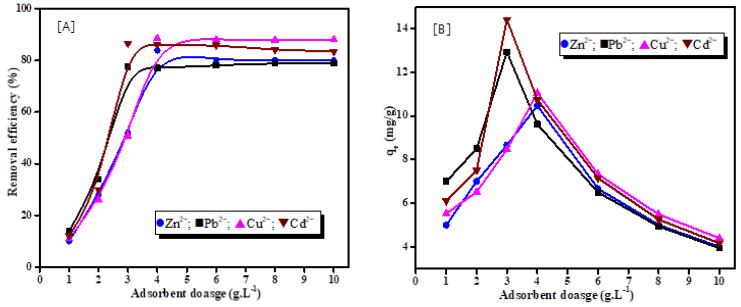
Effect of adsorbent dosage on heavy metal ions removal efficiency (**A**), adsorption capacity of heavy metal ions on synthesized zeolite (**B**).

The effect of the adsorbent dose on the removal efficiency and adsorption capacity of heavy metal ions by synthetic zeolite is presented in Figure 6. The influence of adsorbent dosage was investigated by varying the adsorbent dosage from 1 gL^−1^ to 10 gL^−1^ in 25 mL of each metal solution ($C_{0}$ = 50 mg·L^−1^). Figure 6A demonstrates that the removal efficiency of metal ions increases with increasing the dosage from 1 to 4 gL^−1^, and with the further increases in the adsorbent dosage, the removal efficiency remains nearly constant. At an adsorbent dose of 4 gL^−1^, the highest uptake capacities for Zn^2+^ and Cu^2+^ were found to be 10.475 and 11.06 mg·g^−1^, respectively. At an adsorbent dosage of 3 gL^−1^, the absorption capacities for Pb^2+^ and Cd^2+^ were found to be 12.93 and 14.42 mg·g^−1^, respectively, as presented in Figure 6B.

It has been noted that when the adsorbent dosage increases from 1 to 4 gL^−1^, the adsorption capacity increases rapidly, with a significant drop at a dosage of 4 to 10 gL^−1^. These results are explained by the fact that at a lower adsorbent dosage, the binding sites of adsorbent are completely utilized by the interference between the sorbent and the solute [62]. At high dosages of synthesized zeolite (≥4 gL^−1^), the accessible active sites for heavy metal adsorption were not completely occupied (lack of metal ions in compared with binding sites in the solution). Particle aggregation may be the reason for the low adsorption capacity, which may decrease the overall surface area of the adsorbent and decrease the diffusion rate [73].

#### 3.2.3. Effect of Contact Time

**Figure 7:**
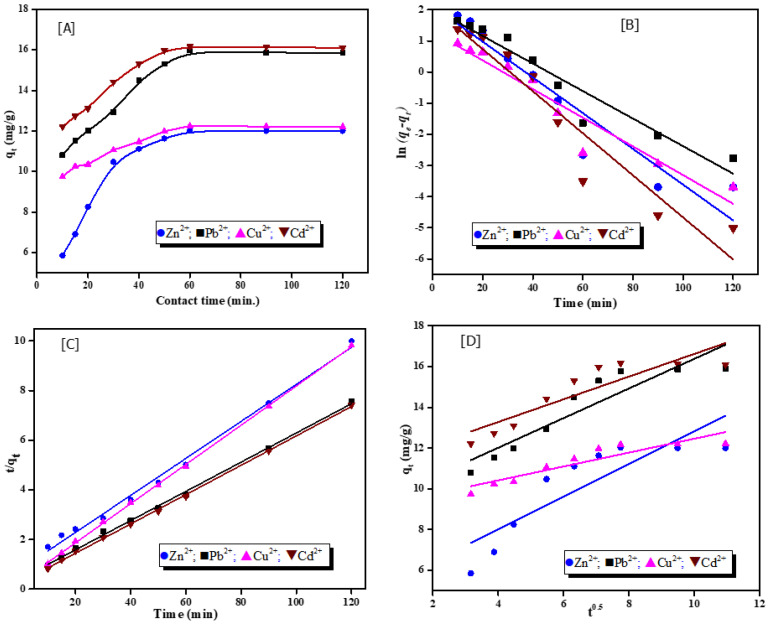
Effect of contact time on Zn^2+^, Pb^2+^, Cu^2+^ and Cd^2+^ adsorption capacity (**A**); Pseudo-first-order (**B**); Pseudo-second-order (**C**); and Intra-particle diffusion (**D**). (Adsorbent dosage = 4 gL^−1^, temperature = 20 °C, initial concentration of metal ions = 50 mgL^−1^ and reaction time = 10–120 min.).

Contact time is a crucial parameter because it represents the adsorption kinetics of an adsorbent at a specific starting concentration of the adsorbate. Figure 7A shows that the adsorption capacities of Zn^2+^, Pb^2+^, Cu^2+^, and Cd^2+^ metal ions increase sharply during the first 30 min. of contact time. In this stage, the adsorption capacities of metal ions grows so quickly due to the presence of unoccupied binding sites and the large zeolite surface area being exposed for metal adsorption [62,63]. After 60 min., Zn^2+^, Pb^2+^, Cu^2+^, and Cd^2+^ uptake capacity reached 12.025, 15.96, 12.24, and 16.17 mg·g^−1^, respectively, as a consequence of the saturation of adsorption sites on the adsorbent surface. This may have been caused by the formation of hardly soluble silicates on the zeolite surface, indicating that the subsequent adsorption became slower and the mechanism causing the adsorption may change [19]. Therefore, in the subsequent adsorption tests, the contact time of 60 min. was recommended and used to give the highest removal efficiency of heavy metal ions.

The obtained kinetic data are a critical aspect in the evaluation of sorption process as a unit operation. In current study, the adsorption kinetics and rate-controlling step of a pseudo-first-order Equation (13) [74], a pseudo-second-order Equation (14) [75], and an intra-particle diffusion model Equation (15) [76] have been evaluated to fit the experimental data and can be presented as follows:

Pseudo-first-order model:

(13) $$\ln\;(q_{e}-q_{t})=\ln\;q_{e}-k_{1}$$

Pseudo-second-order model:

(14) $$\frac{t}{q_{t}}=\frac{1}{k_{2}q_{e}^{2}}+\frac{t}{q_{e}}$$

Intra-particle diffusion model:

(15) $$q_{t}=K_{DM}\;t^{0.5}+C$$

where $q_{e}$ and $q_{t}$ (mg·g^−1^) are the adsorbed amounts of heave metal ions at the equilibrium and the reaction time (min), respectively, $k_{1}$ (min^−1^), $k_{2}$ (g·mg^−1^·min^−1^), and $K_{\mathrm{DM}}$ (mg·g^−1^·min^1/2^) are the rate constants of the different kinetic models and C is the thickness of the boundary layer. The slope and intercept of the linear plot of ln ($q_{e}$ − $q_{t}$) vs. *t* was used to compute $k_{1}$ and $q_{e\;cal}$, respectively, for the pseudo-first order model (Figure 7B). The equilibrium adsorption quantity $q_{e\;cal}\;$ and the pseudo-second-order rate parameters ($k_{2}$) were calculated from the slope and intercept of the plot of $t/q_{t}$ against *t* (Figure 7C). The intra-particle diffusion rate constant $k_{\mathrm{md}}$ and correlation coefficient R^2^ were calculated from the straight-line plots of $q_{t}\;\mathrm{versus}\;t^{0.5}\;$(Figure 7D).

Table 3 summarizes the parameters of the related kinetics models. It has been noted that the pseudo-second-order model provides a superior explanation for the sorption kinetic data, and it has the highest R^2^ value (R^2^ > 0.99), compared with the other models. Moreover, the equilibrium adsorption capacity $q_{e\;cal}$ values computed using the pseudo-second-order model agree more closely with the experimental $q_{e\;exp}$ values. Hence, it can be deduced that the pseudo-second-order adsorption mechanism is prevalent for the adsorption of metal ions onto zeolite, suggesting that the chemisorption was mainly the rate-controlling step, and the adsorption mechanism may be influenced by valence forces through sharing electrons between the adsorbate and the adsorbent as a rate-determining step, rather than mass transfer [63,77].

#### 3.2.4. Effect of Initial Concentration

**Figure 8:**
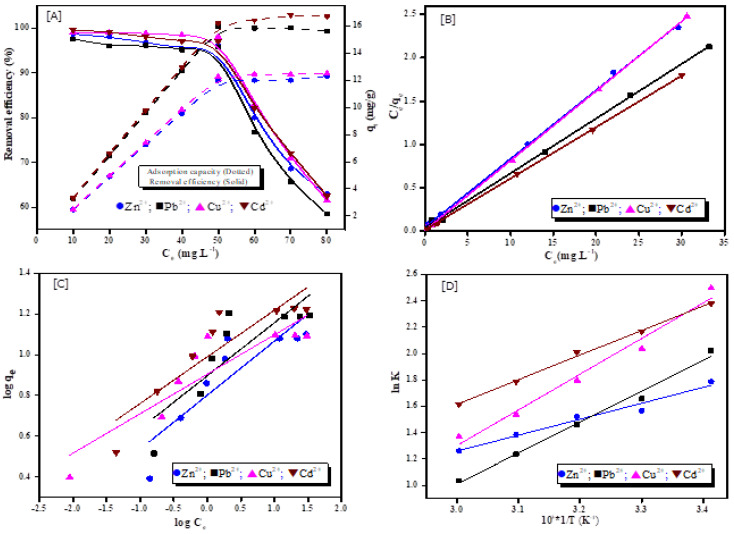
Effect of initial concentration on adsorption of Zn^2+^, Pb^2+^, Cu^2+^ and Cd^2+^ onto adsorbent (**A**), Langmuir model (**B**), Freundlich model (**C**), and adsorption thermodynamic model (**D**). (Adsorbent dosage = 4 gL^−1^, adsorption temperature = 20–60 °C, initial concentration of metal ions = 10–80 mg·L^−1^, and reaction time = 60 min).

The effect of the initial concentration of heavy metal ions on the adsorption efficiency of zeolite was investigated using different concentrations from 10 to 80 mg·L^−1^ at an adsorbent dosage of 4 gL^−1^ and the obtained results are presented in Figure 8A. It has been noted that when the initial concentration increases, the effectiveness of the metal ion removal clearly declines. However, the removal efficiency of Zn^2+^, Pb^2+^, Cu^2+^, and Cd^2+^ increase to their highest level at 96.2%, 95.78%, 97.98%, and 97.02%, respectively, when the initial concentration increases from 10 to 50 mgL^−1^ at the adsorption temperature of 20 °C. Thereafter, it remains stable with little change at any concentration more than 50 mgL^−1^, due to the small number of free adsorption sites on the adsorbent surface [63]. Heavy metal ions in the solution interact directly with the active binding site under initial concentration conditions, which increases the driving force of the metal ions adsorbed on the surface of the adsorbent material. The fact that the adsorption capacity remains steady as the initial concentration increases could be attributed to the metal ions progressively occupying binding sites until saturation is achieved [62]. These results verify that the number of accessible sites on the synthesized zeolite is a crucial factor in the efficiency of the removal of Zn^2+^, Pb^2+^, Cu^2+^, and Cd^2+^ through an adsorption process.

In order to understand the equilibrium adsorption isotherms of Zn^2+^, Pb^2+^, Cu^2+^, and Cd^2+^ onto synthesized zeolite, several models have been proposed, such as Freundlich, Langmuir, and Temkin, which are expressed in Equations (16)–(18).

Langmuir isotherm equation:

(16) $$\frac{C_{e}}{q_{e}}=\frac{1}{q_{max}\;k_{L}}+\frac{C_{e}}{q_{e}}$$

Freundlich isotherm equation:

(17) $$logq_{e}=logK_{F}+\frac{1}{n}\;logC_{e}$$

Temkin isotherm equation:

(18) $$q_{e}=B_{T\;}ln\;k_{T}+B_{T\;}lnC_{e}$$

where $C_{e}$ (mg·L^−1^) is the concentration of metal ions at the equilibrium, $q_{e\;}\mathrm{and}\;q_{max}$ (mg·g^−1^) are the quantity of metal ions adsorbed at the equilibrium and maximum saturated adsorption amount for a monolayer of adsorbent. $k_{L}$ (Lmg^−1^) is the Langmuir equilibrium adsorption constant which is computed from the linear plots of $C_{e}/q_{e}$ vs. $C_{e\;}$ as presented in Figure 8B. $k_{F}$ (mg·g^−1^) and *1/n* are the Freundlich adsorption isotherm constant, which denotes the adsorption capacity of the adsorbent, and the heterogeneity of the adsorption process, respectively, which are computed from the linear plots of lg$q_{e}$ vs. lg$qC_{e}$ as presented in Figure 8C. $K_{T}$ (L·g^−1^) and B_T_ (KJ·mol^−1^) are Temkin constants related to the heat of adsorption and the equilibrium related to binding energy, respectively.

Table 4 illustrates the fitting of the equilibrium data determined by related equations. According to this table, the Langmuir isotherm has the highest correlation coefficients (R^2^) of 0.9991, 0.9984, 0.9997, and 0.9996 for Zn^2+^, Pb^2+^, and Cu^2+^, and Cd^2+^, respectively, compared with other adsorption isotherm models. According to these values, the Langmuir model provides a better fit to the experimental data and the nature of the adsorption of the four different metal ions on the adsorbent is the most consistent with Langmuir assumptions.

The dimensionless separation factor $\left(R_{L}\right)$ is used to predict if the adsorption system is favorable or not, and is expressed using Equation (19) [35].

(19) $$R_{L}=\frac{1}{1+K_{L}C_{0}}$$

where $C_{0}$ is the initial concentration of metal ions (mg·L^−1^) and $k_{L}$ is the Langmuir constant. The $R_{L}$ values calculated for Zn^2+^, Pb^2+^, Cu^2+^, and Cd^2+^ ions are 0.00720, 0.00753, 0.0016, and 0.0032, respectively, which can be expressed as 0 <$\;R_{L}$ < 1. These values suggest that the adsorption process of the metal ions onto the adsorbents is favorable in all of the conditions investigated [66,78]. The spontaneity of the adsorption response is reflected by the Langmuir constant $K_{L}$, which is a characteristic parameter related to the binding energy of the solute and adsorbent. With more $K_{L}$, the adsorption process will be more spontaneous and produce a product that is more stable and has a larger adsorption capacity. The value of $K_{L}$ in this investigation is low, suggesting that the four metal ions adsorption product on the adsorbent is unstable, indicating that the Zn^2+^, Pb^2+^, Cu^2+^, and Cd^2+^ adsorbed on the adsorbent might be simply desorbed [62]. Hence and according to the obtained results, the adsorption of the metal ions Zn^2+^, Pb^2+^, Cu^2+^, and Cd^2+^ occurs on the surface of the material via monolayer coverage (chemical) with homogeneous binding points, and a similar adsorption energy. As a result, the Langmuir isotherm model is considered to be suitable for the explanation of the adsorption process.

Table 5 presents a comparison between the adsorption capacity results of zeolite adsorbents for heavy metal ions obtained in this study and that of other studies. It was noted that the synthesized zeolite based on SASR had a similar adsorption capacity to other adsorbents and exhibits a good adsorption capacity for several types of metals present in wastewaters.

### 3.3. Adsorption Thermodynamics

The related thermodynamic parameters for the influence of temperature on heavy metal ions adsorption mechanism, according to thermodynamic theory, were calculated using the following equations. Entropy (∆*S*^0^), enthalpy (∆*H*^0^), and free energy change (∆*G*^0^) were computed using the flowing Equations (20)–(23).

(20) $$\Delta G^{0}=-RT\;lnK_{c}$$

(21) $$K_{d}=\frac{q_{e}}{C_{e}}$$

(22) $$\Delta G^{0}=\Delta H^{0}-T\Delta S^{0}$$

(23) $$lnK_{L}=\frac{\Delta S^{0}}{R}-\frac{\Delta H^{0}}{RT}$$

where $K_{L}$ is the Langmuir equilibrium constant, R is the universal gas constant (8.314 J mol^−1^ K^−1^), and T is the temperature of the reaction (K). The values of ∆*H*^0^ and ∆*S*^0^ can be obtained as the slope and intercept from a linear plot between lnk versus 1/T (Figure 8D). The obtained results and the corresponding thermodynamic data are presented in Table 6, which shows that the adsorption process is of exothermic nature because the obtained ∆*H*^0^ values from 293 to 333 °K are negative. Moreover, the decrease in K_L_ and change of the absolute |ΔG0| value as the temperature increases indicates that the adsorption process becomes unfavorable at higher temperature. The negative values of (∆*G*^0^) and standard entropy change (ΔS^0^) indicate that spontaneous and effective adsorption of Zn^2+^, Pb^2+^, Cu^2+^, and Cd^2+^ ions onto synthesized zeolite adsorbent are more favorable at low temperatures, along with a reduction in the degree of randomness at the solid–liquid interfaces during the adsorption process [78,84].

### 3.4. Adsorption Mechanism of Heavy Metal Ions upon Synthesized Zeolite

**Figure 9:**
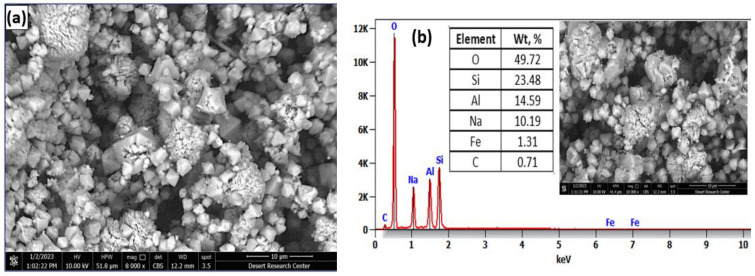
SEM image of synthesized zeolite (8000× magnification) (**a**) and the EDS element analysis (10,000× magnification) (**b**).

In order to study the mechanism of heavy metal ion adsorption by synthesized zeolite, the surface morphology of the synthesized zeolite was investigated using the SEM-EDX analysis, the results of which are presented in Figure 9. The micrographs in Figure 9a reveal that the synthesized zeolite has a cubic crystal structure with a smooth surface and an average diameter of 5–10 μm, as well as acicular structures with a few tiny pores [19,26]. This structure can be attributed to the formation of zeolite based on the alumino-silicate glass phase. The EDX spectrum presented in Figure 9b shows that the synthesized zeolite is composed mainly of silicon, aluminium, oxygen, sodium, and a small amount of iron was also detected.

**Figure 10:**
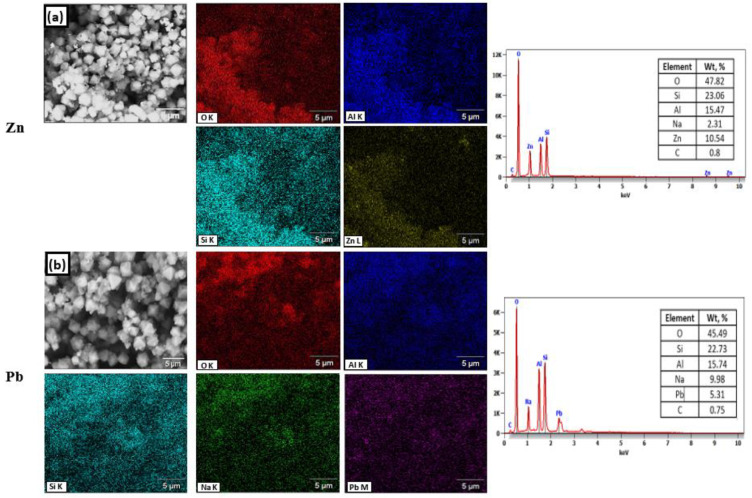
SEM image of the synthesized zeolite at SASR-Kaolin weight ratio of 1:1.5: (**a**) mapping of Zn^2+^ loaded, O, Al, Si, Zn, and EDS element analysis, (**b**) mapping of Pb^2+^ loaded, O, Al, Si, Na, Pb, and EDS element analysis.

After the adsorption process, it was noted that the crystalline structure of the synthesized zeolite remains almost unchanged, and the surface becomes rough after the adsorption of Zn^2+^ and Pb^2+^ ions as shown in Figure 10. Figure 10a shows the structure of zeolite after the loading of Zn^+2^ ions. Based on SEM-elemental mapping and EDS analysis, it is clear that the loaded zeolite is composed of O, Al, Si, and Zn with contents of 47.82, 15.47, 23.06, and 10.54 wt.%, respectively, with a small amount of Na (2.31 wt.%) compared to the elemental zeolite composition (Figure 9b). This suggests that ion exchange is a factor in the adsorption mechanism [78]. Figure 10b shows the structure of zeolite after the loading of Pb^+2^ ions. It is clear that the SEM-based elemental mapping combined with the EDS analysis of the loaded zeolite consists of O, Al, Si, Na, and Pb with contents of 45.49, 15.74, 22.73, 9.98, and 5.31 wt.%, respectively.

**Figure 11:**
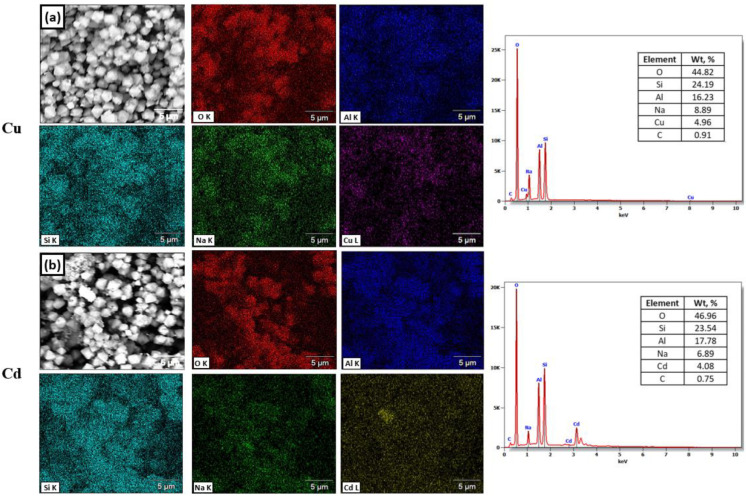
SEM image of the synthesized zeolite at SASR-Kaolin weight ratio of 1:1.5: (**a**) mapping of Cu^2+^ loaded, O, Al, Si, Na, Cu, and EDS element analysis, (**b**) mapping of Cd^2+^ loaded, O, Al, Si, Na, Pb, and EDS element analysis.

The EDS maps and spectrum elements analysis of Cu^2+^ and Cd^2+^ ions adsorbed on the synthesized zeolite are presented in Figure 11. Figure 11a shows that the surface of synthesized zeolite has an irregular, rough surface and the content of O, Al, Si, Na, and Cu were 44.82, 16.23, 24.19, 8.89, and 4.96 wt.%, respectively. These analyses indicate that surface adsorption and precipitation are most likely the predominant mechanisms by which Cu^2+^ ions can be absorbed by zeolite [73]. On the other hand, the Cd-loaded zeolite shown in Figure 11b had a O, Al, Si, Na, and Cd content of 46.96, 17.78, 23.54, 6.89, and 4.08 wt.%, respectively. The characteristic analysis demonstrates that the ion exchange in the few places available for this process, followed by an adsorption complexation reaction, are the main adsorption mechanisms of the removal of Cd^2+^ ions [62].

**Figure 12:**
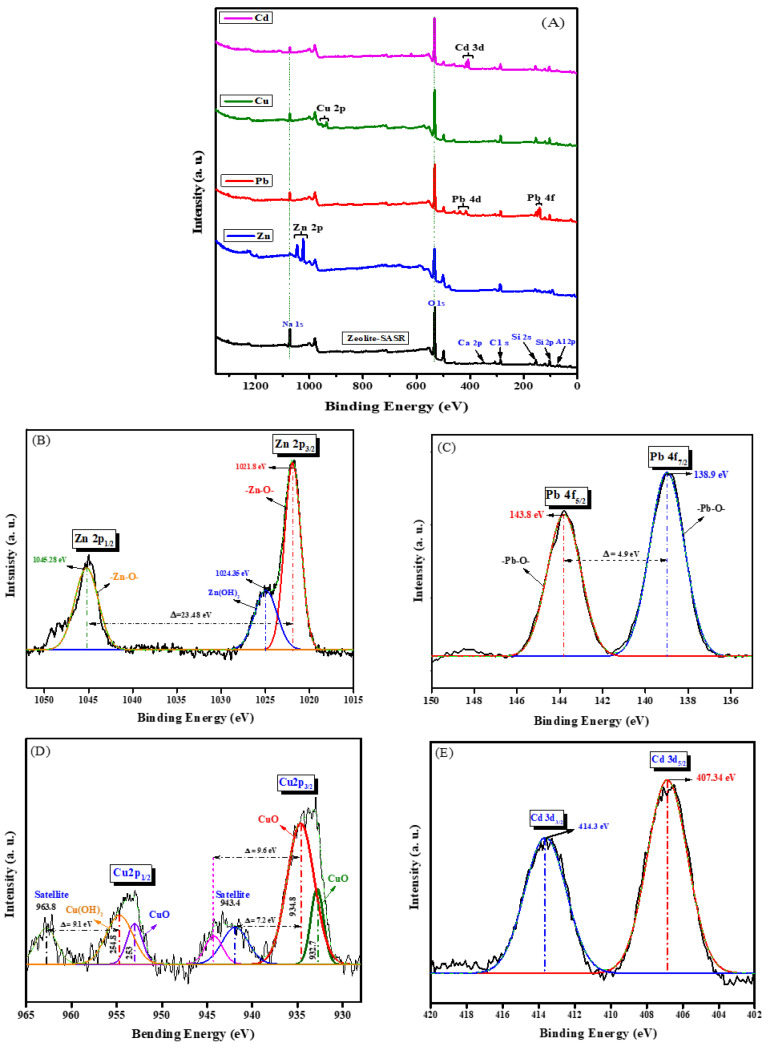
XPS wide scan of synthesized zeolite before and after Zn, Pb, Cu, and Cd adsorption: Full range (**A**), High-resolution XPS spectra of Zn2p (**B**), Pb4f (**C**), Cu2P (**D**), and Cd3d (**E**).

To gain a deeper understanding of the adsorption mechanisms, X-ray photoelectron spectroscopy (XPS) was employed to identify the surface chemistry of the synthesized adsorbent and the nature of its interaction with metal ions. Figure 12A shows the complete spectra of the synthesized zeolite surface before and after adsorption. The major elements displayed on the XPS spectra agree with the EDS findings as presented in Figure 10 and Figure 11.

As shown in Figure 12B, after Zn^2+^ adsorption, it was confirmed that the new peaks at approximately 1021.8 and 1045.3 eV in the high-resolution were assigned to Zn2p_3/2_ and Zn2p_1/2_, respectively. Moreover, the peak of Na1s at approximately 1073.2 eV significantly decreased to the lowest level, indicating that Zn^2+^ was successfully adsorbed onto the surface of the adsorbent via ion exchange [85]. Meanwhile, the appearance of a peak at approximately 124.35 eV indicates that Zn^2+^ can further directly react in a few places available with OH^−^ resulting in the formation of precipitation compounds (Zn(OH)_2_) on the surface of synthesized zeolite. After Pb^2+^ adsorption, there are two distinct peaks in the range of 138.9–143.8 eV as shown in Figure 12C. The peak at approximately 138.9 eV is attributed to Pb4f_7/2_, and the peak near 143.8 eV was assigned to Pb4f_5/2_. The obtained results indicate that the adsorption of Pb^2+^ to the surface of the adsorbent was affected by surface precipitation or complexation adsorption mechanism [19].

Peaks observed near 933.1 and 953.7 eV on the binding energy of the high-resolution scale were assigned to Cu2p_3/2_ and Cu2p_1/2_ photoelectrons, respectively, as seen in Figure 12D. Additionally, the two shakeup satellites are easily observed in the samples at approximately 943.2 and 963.8 eV, which are at a higher binding energy of approximately 7.2 and 9.6 eV above the main Cu2p_3/2_, and at about 9.1 eV above the Cu2p_1/2_ peaks, respectively. The presence of strong satellite characteristics of Cu2p excludes the possibility of a Cu_2_O phase [85]. Meanwhile, there was no difference in the peak intensity of Na1s before and after the adsorption experiments, proposing that the Cu^2+^ ions were attached to the surface of the adsorbent by surface precipitation or complexation adsorption mechanisms [8], which aligns with the SEM-EDX results in Figure 11a. Figure 12E shows the high resolution XPS spectrum of Cd3d, which reveals the binding energies of Cd3d_5/2_ at approximately 407.34 eV and Cd3d_3/2_ centered at 414.3 eV. The obtained results demonstrate that the Cd^2+^ ions were attached to the surface of the synthesized zeolite by surface precipitation or complexation adsorption mechanisms [19].

**Figure 13:**
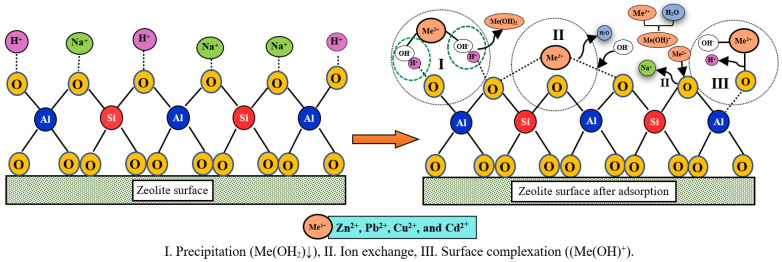
The proposed adsorption mechanism of heavy metal ions on synthesized zeolite.

According to EDX and XPS observations, the proposed absorption mechanism in Figure 13 shows that the adsorption of Zn^2+^, Pb^2+^, Cu^2+^, and Cd^2+^ on zeolite likely can be divided into three major phenomena: ion exchange, adsorption, and precipitation as mentioned in [86]. The cations exchange is the key mechanism for the absorption of Zn^2+^ and Cd^2+^ from aqueous solutions. This process can be represented using the following equations:

(Zeolite-SASR)Na^2+^ + Me^2+^ → (Zeolite-SASR)Me^2+^ + Na^2+^ (24)

Table 7 shows the significant difference in the binding energies of O1s peaks (532.3 eV) before and after adsorption of the heavy metal ions, provided that the surface of synthesized zeolite was rich in metal oxide functional groups and active adsorption sites, and the existence of hydroxyl groups in the adsorbent promoted the metal ions adsorption. Additionally, the complexes were created by sharing a long pair of electrons in the O atom between O and metal ions which may be responsible for raising the binding energy of O1s [63]. As a result, there was a drop in the density of the electron cloud surrounding the O atom, and an increase in binding energy, corresponding closely to the findings in Section 3.2.1.

### 3.5. Treatment of EGPC Industrial Wastewater Sample

The targeted levels of heavy metal ion concentrations prior to and during adsorption using synthesized zeolite are shown in Table 8. The concentrations are compared with World Health Organization (WHO) recommendations for wastewater drainage. In terms of drainage water regulations, zinc, lead, copper, and cadmium are the most crucial elements present in the wastewater sample taken from the Egyptian General Petroleum Corporation (EGPC). Within the context of the experimental setting, the residual values are consistently below the detection limits, with the exception of Mn (2.1 mgL^−1^) and Fe (3.1 mgL^−1^) for the first cycle. However, in the fourth adsorption cycle, the residual heavy metal ion concentrations are in accordance with the WHO guidelines for wastewater disposal in the marine environment with high removal efficiency [87]. When the number of circulations is increased (5th and 6th cycles), the amount of heavy metal ions in the effluent also goes up. As a result, when the pollutants are enriched to a specific level, the adsorbent needs to be cleaned in order to achieve closed recycling. Finally, the synthesized zeolite can be utilized to safeguard the industrial wastewater drainage system.

## 4. Conclusions

Synthesized zeolite is an effective adsorbent for the removal of Zn^2+^, Pb^2+^, Cu^2+^, and Cd^2+^ heavy metal ions from industrial wastewater, based on readily accessible and low-cost raw materials. High-crystallin (85.21%) and cost-effective zeolite has been successfully synthesized based on a mixture of SASR-kaolin using the alkaline fusion-hydrothermal method. It was noted that the weight ratio of SASR- kaolin mixture of 1:1.5 gives the best composition and properties of the synthesized zeolite. The optimal conditions for the synthesized zeolite were a fusion temperature of 600 °C, a 1:3 wt. ratio (SASR- kaolin)-NaOH, a 1:4 solid-liquid ratio, an 80 °C crystallization temperature, and a 24 h crystallization time.

It was noted that the adsorption process was of an exothermic, pH-dependent, spontaneous nature. Significant adsorption occurs when the pH is 7.0 for Zn and Cd ions and 6.0 for Pb and Cu ions. The adsorption process was found to follow a pseudo-second-order kinetic model and a Langmuir isotherm model. The maximum monolayer adsorption capacities of Zn^2+^, Pb^2+^, Cu^2+^, and Cd^2+^ ions onto zeolite at 20 °C were 12.025, 15.96, 12.247, and 16.17 mg·g^−1^, respectively, showing a significantly greater removal efficiency, which aligns with the previously obtained results. The main mechanisms controlling the process of the removal of these metal ions from an aqueous solution by synthesized zeolite is proposed to be either surface adsorption, precipitation, or ion exchange.

The obtained results prove that the synthesized zeolite is effective in removing heavy metal ions from the selected wastewater sample. Meanwhile, the adsorption efficiency decreases with progressive recycling steps (for four times) due to saturation of adsorption sites on the surface of the adsorbent.

## Figures and Tables

**Table 1:** Overall chemical composition of the sulfuric acid solid residue (SASR) before and after the pre-treatment processes.

Component	SASR (Wt.%)	Magnetic and Dia. HCl Leaching (Wt.%)	−45 µm Kaolin (Wt.%)	Calcined Mixture
SiO_2_	26.57	59.34	47.12	49.54
Al_2_O_3_	5.81	11.75	36.66	27.81
CaO	10.2	2.42	0.32	1.42
K_2_O	0.06	0.81	0.13	0.63
Fe_2_O_3_	18.93	2.37	0.63	1.05
Na_2_O	0.01	0.18	0.07	14.71
TiO_2_	0.38	0.11	1.32	0.65
MgO	6.83	2.61	0.26	1.14
MnO	0.101	0.32	0.22	0.32
P_2_O_5_	3.61	0.29	0.35	0.21
Others	9.62	2.05	0.42	0.37
L.O. I	17.88	17.75	12.5	2.15

**Table 2:** The surface characteristics of the synthesized zeolite.

Sample	SASR-Kaolin Weight Ratio	Surface Area, (S_BET_ m^2^/g)	Total Pore Volume, (cm^3^/g)	Average Pore Diameter, (nm)
Zeolite	0:1	75.46	0.112	6.63
	1:2	473.54	0.226	8.41
	1:1.5	465.06	0.207	8.23
	1:1	371.42	0.166	7.96
	2:1	193.18	0.153	7.15
	1:0	62.84	0.109	4.24

**Table 3:** A comparison for rate constants of pseudo-first-order, pseudo-second-order and intraparticle diffusion kinetic models calculated from experimental data.

Metal Ions	Experimental Data	Pseudo-First-Order			Pseudo-Second-Order			Intra-Particle Diffusion		
	$\;q_{e,\;\;\;exp}/\;$ $\left(mg\cdot g^{-1}\right)$	$\;q_{e,\;\;\;cal\;}/\;$ $\left(mg\cdot g^{-1}\right)$	K_1_/ (min^−1^)	R^2^	$\;q_{e,\;\;\;cal}/\left(mg\cdot g^{-1}\right)$	K_2_/ (g·mg^−1^· min^−1^)	R^2^	$K_{DM}/\;$(mg·g^−1^·min^1/2^)	C	R^2^
Zn	12.025	8.38	0.066	0.9052	13.40	0.0071	0.995	0.81	0.4808	0.752
Pb	15.963	7.73	0.043	0.9301	16.98	0.0086	0.998	0.727	9.106	0.854
Cu	12.247	3.66	0.046	0.9235	12.66	0.0221	0.999	0.341	9.049	0.848
Cd	16.17	8.08	0.068	0.916	16.89	0.0135	0.997	0.557	11.05	0.801

**Table 4:** Adsorption isotherm modeling for the metal ions adsorption onto synthesized Zeolite.

Metal Ions	Experimental Data		Langmuir			Freundlich			Temkin		
	${\;q}_{e,\;\exp}/$ $\left(\mathrm{mg}\cdot {\;g}^{-1}\right)$	${\;q}_{e,\;\mathrm{cal}}/$ $\left(\mathrm{mg}\cdot {\;g}^{-1}\right)$	K_L_/ (L·mg^−1^)	R_L_	R^2^	K_F_/ (mg·g^−1^)/(mg L^−1^)^1/n^	$\frac{1}{n}$	R^2^	$B_{T}$ $J\cdot {\mathrm{mol}}^{-1}$	$K_{T}$(L·m^−1^)	R^2^
Zn	12.025	12.65	1.746	0.00720	0.9991	6.33	0.2646	771.0	1.81	59.28	0.837
Pb	15.963	15.84	1.632	0.00753	0.9984	7.89	0.261	0.719	2.261	60.57	0.743
Cu	12.247	12.46	7.425	0.0016	0.9997	8.02	0.1935	0.814	1.305	230.23	0.828
Cd	16.17	16.83	3.88	0.0032	0.9996	9.76	0.2284	0.819	2.14	209.2	0.871

**Table 5:** A comparison between the different types of cost-effective adsorbents for adsorption of heavy metal ions.

Adsorbent	Adsorption Capacity (mg·g^−1^)	Temp./°C	Ref.
Zeolite-based OSA	156.74 (Cu^2+^), 224.72 (Pb^2+^), 118.34 (Cd^2+^)	20	[8]
Zeolite-based Fly ash (FA)	25.64 (Pb^2+^), 34.24 (Zn^2+^)	25	[9]
Zeolite-Modified oil shale ash (MOSA)	34.96 (Pb^2+^), 29.41 (Zn^2+^)	25	[9]
Zeolite-based coal fly ash	26.25 (Cd^2+^)	-	[66]
Zeolite–based OSA	70.58 (Pb^2+^), 95.6 (Cd^2+^)	30	[61]
Hydrothermally modified fly ash	87.72 (Cd^2+^), 56.497 (Cu^2+^)	-	[79]
Hydrothermally modified CFB fly ash (HM-CFB-FA)	183.7 (Cd^2+^)	-	[62]
Low temperature-NaOH modified fly ash	75.01 (Cd^2+^)	30	[63]
CFA-NaOH	76.7–137.1 (Cu^2+^)	30–60	[80]
Fly ash zeolite X	420.2(Pb^2+^), 90.86 (Cu^2+^), 97.78 (Cd^2+^)	25	[81]
Zeolite clinoptilolite	9.03 (Cu^2+^)	25	[82]
Silver-modified clay adsorbent (Ag/clay)	97.8 (Zn^2+^), 97 (Pb^2+^), 96.4 (Cu^2+^)	-	[78]
Hydrothermally modified fly ash	65.75 (Pb^2+^), 56.06 (Cu^2+^), 52.12 (Cd^2+^)	25	[83]
Zeolite–based SASR with Kaolin at 1:1.5 weight ratio	12.025 (Zn^2+^), 15.96 (Pb^2+^), 12.247 (Cu^2+^), 16.17 (Cd^2+^)	20	Current study

**Table 6:** Values of thermodynamic parameters for Zn^2+^, Pb^2+^, Cu^2^+ and Cd^2+^ adsorption upon the synthesized zeolite.

Metal Ions	$\Delta H^{0}$	$\Delta S^{0}$	$\;\;\Delta G^{0}\left(KJ\cdot mol^{-1}\right)$					R^2^
	$\left(KJ\cdot mol^{-1}\right)$	$\left(J\cdot mol^{-1}K^{-1}\right)$	293 °K	303 °K	313 °K	323 °K	333 °K	
Zn	−10.047	−19.66	−4.352	−3.946	−3.957	−3.714	−3.489	0.971
Pb	−19.491	−50.06	−4.929	−4.172	−3.809	−3.322	−2.867	0.991
Cu	−22.453	−56.53	−6.078	−5.129	−4.676	−4.128	−3.789	0.998
Cd	−15.466	−32.97	−5.791	−5.456	−5.227	−4.791	−4.472	0.996

**Table 7:** Binding energy and contents of elements on synthesized zeolite surface.

Metal Ions	Binding Energy (eV)										Surface Atomic Composition (%)							
	O1s	C1s	Si2p	Al2p	Na1s	Zn2p	Pb4f	Cu2p	Cd3d	O1s	C1s	Si2p	Al2p	Na1s	Zn2p	Pb4f	Cu2p	Cd3d
Zeolite-SASR	532.3	285.9	103.3	75.11	1073	–	–	–	–	50.9	13.1	15.9	8.11	8.63	–	–	–	–
Zn-loaded	533.1	286.9	104.3	75.69	1073.2	1023.1	–	–	–	45.2	31.4	10.6	4.85	1.04	6.91	–	–	–
Pb-loaded	532.4	285.4	103.1	75.51	1073.1	–	139.9	–	–	49.6	15.5	18.5	7.84	5.81	–	2.72	–	–
Cu-loaded	532.5	285.8	103.2	75.25	1073	–	–	935.5	–	52.3	12.3	19.7	5.77	6.35	–	–	3.46	–
Cd-loaded	533.1	286.5	104.3	76.12	1073.1	–	–	–	407.2	52.4	14.8	18.4	7.91	3.86	–	–	–	2.52

**Table 8:** Applying the synthesized zeolites for removing heavy metal ions from EGPC industrial wastewater sample.

Metals		Cd	Cr	Cu	Mn	Pb	Fe	Ni	Zn	V
$C_{0}$(mgL^−1^)		6.127	7.016	10.294	8.152	11.493	8.219	0.002	17.051	0.01
MLD _WHO_		0.01	0.01	1	0.1	0.01	1.5	0.1	1	0.002
**Number of Cycles**	1	U.D	U.D	U.D	2.1	U.D	3.1	U.D	U.D	U.D
	2	U.D	U.D	U.D	0.6	U.D	1.1	U.D	U.D	U.D
	3	U.D	U.D	U.D	0.05	U.D	U.D	U.D	U.D	U.D
	4	U.D	U.D	U.D	U.D	U.D	U.D	U.D	U.D	U.D
	5	0.08	0.05	1.3	2.12	0.51	2.5	0.054	1.66	0.027
	6	0.45	2.01	1.7	7.12	1.07	4.5	0.098	3.87	0.089

U.D: Under detection limit; MLD _WHO_: Maximum limits for wastewater disposal according to WHO.
